# Comparison of participants and non-participants to the ORISCAV-LUX population-based study on cardiovascular risk factors in Luxembourg

**DOI:** 10.1186/1471-2288-10-80

**Published:** 2010-09-07

**Authors:** Ala'a Alkerwi, Nicolas Sauvageot, Sophie Couffignal, Adelin Albert, Marie-Lise Lair, Michèle Guillaume

**Affiliations:** 1 Centre de Recherche Public de la Santé, Centre d'Etudes en Santé, Grand-Duchy of Luxembourg; 2 School of Public Health, University of Liège, Belgium

## Abstract

**Background:**

Poor response is a major concern in public health surveys. In a population-based ORISCAV-LUX study carried out in Grand-Duchy of Luxembourg to assess the cardiovascular risk factors, the non-response rate was not negligible. The aims of the present work were: 1) to investigate the representativeness of study sample to the general population, and 2) to compare the known demographic and cardiovascular health-related profiles of participants and non-participants.

**Methods:**

For sample representativeness, the participants were compared to the source population according to stratification criteria (age, sex and district of residence). Based on complementary information from the "medical administrative database", further analysis was carried out to assess whether the health status affected the response rate. Several demographic and morbidity indicators were used in the univariate comparison between participants and non-participants.

**Results:**

Among the 4452 potentially eligible subjects contacted for the study, there were finally 1432 (32.2%) participants. Compared to the source population, no differences were found for gender and district distribution. By contrast, the youngest age group was under-represented while adults and elderly were over-represented in the sample, for both genders. Globally, the investigated clinical profile of the non-participants was similar to that of participants. Hospital admission and cardiovascular health-related medical measures were comparable in both groups even after controlling for age. The participation rate was lower in Portuguese residents as compared to Luxembourgish (OR = 0.58, 95% CI: 0.48-0.69). It was also significantly associated with the professional status (*P *< 0.0001). Subjects from the working class were less receptive to the study than those from other professional categories.

**Conclusion:**

The 32.2% participation rate obtained in the ORISCAV-LUX survey represents the realistic achievable rate for this type of multiple-stage, nationwide, population-based surveys. It corresponds to the expected rate upon which the sample size was calculated. Given the absence of discriminating health profiles between participants and non-participants, it can be concluded that the response rate does not invalidate the results and allows generalizing the findings for the population.

## Background

Poor response rate and sample representativeness are major concerns in prevalence public health surveys[1-5]. Nevertheless such studies are based on voluntary participation which depends basically on the subjects' willingness to take part. The literature shows that response rates vary tremendously among surveys and are decreasing in most industrialized countries[6]. In addition, the participation to health surveys requiring physical, anthropometric and blood examination of subjects tends to be even lower in comparison to only-questionnaire surveys[7-9]. Several factors affect the collaboration and participation of subjects, such as population characteristics, cultural attitudes, way of initial contact, length of time needed for full participation, follow-up procedure, legitimacy of the research from the subject's viewpoint, and subject's interest by the research topic[7-9]. Noteworthy, only few published studies drew attention to the real denominator (total eligible sample) to calculate the true participation rate. This makes a comparison of response rates across similar studies quite difficult.

Traditionally, a high response rate is dictated as a rule of thumb of good survey practice. It is considered as an indicator of quality survey estimates, by presuming that a high response rate is associated with a more representative sample and hence with lower bias[10]. Though several studies showed that non-response can yield information with large potential bias[11,12], recent empirical findings, based on relevant methodological studies by Groves and colleagues, suggest however that changes in non-response rates do not necessarily alter survey estimates. The link between non-response rate and bias is indirect and more complex[6]. More and more, there is less support for the hypothesis that low response rate produces systematically high non-response bias estimates[6,13,10-16]. Moreover, the same group of experts in the field advised to anticipate the coverage error by applying a prior well-defined sampling frame so as to retain the value of unbiased sample despite low response rates[6,14,13]. In short, non-response bias is a much more complex phenomenon than mere non-response rates, which needs to be investigated consistently in epidemiological surveys[17]. The literature shows that non response is generally managed in two ways. The first is by using response-enhancement strategies during survey development and data collection. This approach is to influence positively the response rate[14,18], particularly among those initially reluctant, as for example incentives for participation and reminders to non-responders[19-22]. The second approach is the post-survey adjustment of data using weighting techniques to correct for non-response errors[18].

Observation of Cardiovascular Risk Factors in Luxembourg (ORISCAV-LUX) is a nationwide cross-sectional cardiovascular monitoring survey, conducted between November 2007 and January 2009, under the auspices of Luxembourg's Ministry of Health and co-financed by the Ministry of Research. Its major public health objective was to establish baseline information on the prevalence of various potentially modifiable cardiovascular risk factors among the non-institutionalized general adult population, aged 18-69 years. Luxembourg is a small country surrounded by Belgium, France and Germany, with a total population of 493,500 inhabitants (STATEC official estimate, 2009) over an area of about 2,600 km^2^. Luxembourgish people constitute approximately 56.3% of the population, while the well-integrated foreign residents, constituting more than 150 nationalities, are mostly Portuguese (16.2%), French (5.8%), Italians (3.9%) and Belgians (3.4%).

The response rate attained 30% in the ORISCAV-LUX survey. However, the low participation rate does not necessarily result in a selection bias[23,24]. Ideally, a separate investigation on non-participants is recommended to reveal potential outcome differences that might indicate reasons for non-participation and subsequent potential non-participation bias[5,23]. To address this concern, the present paper aimed to 1) investigate the representativeness of the ORISCAV-LUX sample with respect to the general population, and 2) compare the known demographic and cardiovascular health-related characteristics of participants and non participants.

## Methods

### Overview of the survey

The ORISCAV-LUX survey was primarily designed to collect baseline cardiovascular health data from adult subjects aged 18-69 years residing in Luxembourg. Such an epidemiological survey was never undertaken before in this country. Its methodology is described in detail elsewhere[25]. Basically, a representative random sample was drawn from the national health insurance registry, stratified by gender (male and female), age (5-year categories) and geographic districts of residence (Luxembourg, Diekirch and Grevenmacher). With a 98% social coverage rate, the registry is considered as the most complete list of inhabitants available in Luxembourg. The minimal representative sample size was calculated to 1285 subjects to ensure statistical power[26]. However, based on literature review and previous evidence with such multiple-stage population-based studies, a high non-participation rate was expected, including refusal, invalid addresses and non-response. Assuming a response rate of 30% and a proportion of 5% of institutionalized subjects in each stratum, the sample size was augmented to 4496 subjects. The distribution of selected subjects in each stratum was proportional to their distribution in the source population (adult population of 18 to 69 years residents in Luxembourg). Pregnant women, people living in institutions, subjects outside the age range 18-69 years and those deceased before recruitment were excluded. Briefly, full participation included three major stages: (i) self completion questionnaire on demographic and socio-economic status, personal and family history of cardiovascular diseases and relevant cardiovascular risk factors including tobacco and alcohol consumption, physical activity, dietary habits and medication intake; (ii) physical and anthropometric measurements; and (iii) blood, urine and hair tests.

### Measures to increase participation

Intensive efforts were made to increase the response rate, not only during survey preparation but also during the participants' recruitment phase. At kick-off phase, survey publicity, including flyers and media articles, were diffused to provide the selected individuals ample information about the study objectives, relevance to public health policies, ways of participation and participant's rights. A telephone number for inquiries was supplied and the ORISCAV-LUX website was set up. A detailed personal invitation letter with a prepaid reply envelope addressed to the research centre was enclosed with each mailing. The selected subjects were asked to send their approval and phone number for follow-up contact and appointment; otherwise, they were requested to mention the reason of refusal with the purpose of obtaining a prior idea on the reasons of non-participation.

Up to three written invitations (first letter and 2 reminders) were sent at monthly intervals to those who did not respond spontaneously. They were followed by several telephone contacts by skilled personnel, in order to recruit the remaining non-respondents. Those who refused were not contacted again. The field-nurses and phone interviewers were all trained and supplied with detailed manuals. Reluctant cases were followed up by the project manager by means of personal phone contact, to discuss the potential reasons and give them the opportunity to ask further questions about the study. To ensure smooth and well organized data collection, several investigation centers dispatched all over the national territory were prepared to welcome the participants, every morning and occasionally on Saturdays. Home visits were also proposed to those who had transport difficulties related to their health status.

Due to the cultural diversity in Luxembourg, all documents (invitation letter, coupon-answer, consent and questionnaires) were translated into the three most spoken languages: German, Portuguese and English. The translated questionnaires, originally established in French, were independently back-ward translated into French to ensure the validity[27]. Multi-lingual field-nurses and phone interviewers were employed. These measures were taken to increase the potential participation of the minorities. Another method of increasing response rate was providing anonymity to the subject, by using only research identification number and code-bar for each participant's file. A clear sentence was written over the communicated documents, pointing out the approval of the national ethical committee and the national council for private data protection. In addition, mutual benefit theory was applied, aiming to maximize the participation, by supplying the participants with their biological results complemented by the cardiologist notes. In case of abnormal findings, they were advised to consult their family doctor.

### Complementary information

The General Inspectorate of Social Security (IGSS) of Luxembourg manages the national health insurance registry, also called the "medical administrative database", from which the ORISCAV-LUX sample of 4496 subjects was selected. Interestingly, besides information about the name, gender, age group, nationality, professional status and address of every resident in Luxembourg, the register contains hospital discharge and medications consumption data. Therefore, complementary anonymous information from the "medical administrative database" was obtained by means of subsequent official request to the IGSS for both participants (1432 subjects) and non-participants (3018 subjects). The requested data concerned cardiovascular health-related conditions, such as medications intake, hospital admissions and medical measures recorded in year 2006 prior to recruitment. Specifically, the requested information comprised (i) prescribed medications, including anti-diabetics, anti-thrombotics, cardiac therapy, antihypertensive drugs, diuretics, peripheral vasodilatators, vasoprotective agents, beta-blocking agents, calcium channel blockers, agents acting on the renin-angiotensin system and serum lipid reducing agents; (ii) hospitalizations due to chronic conditions, such as malignant neoplasm of the colon, bronchus, lung, prostate, breast, thyroid; mental and behavioral disorders due to use of tobacco, alcohol and nutritional disorders, severe mental retardation, hypertensive diseases, ischemic heart disease, cardiac arrest, cardiac failure, cerebro-vascular disease, atherosclerosis, chronic liver cirrhosis, acute and chronic renal failure, and (iii) other cardiovascular-related medical measures, such as hemodialysis for renal failure, cardiac pacemaker, thyroid gland scintigraphy, cardiac catheterization and coronary angioplasty. These data are indicators of morbidity covering a time period of 12 months prior to the study launch. Information concerning the distribution of the resident population of Luxembourg was available freely from the National Statistics and Economic Studies Service (STATEC).

### Ethical aspects

As long as the additional requested data were anonymous, the participant's consent was not necessary to make the link between data collected through the ORISCAV-LUX survey and the medical administrative database.

### Statistical methods

All statistical analyses were carried out using the SAS 9.2 (^© ^SAS Institute Inc., Cary, NC, USA). Results were presented as numbers and percentages. To study the ORISCAV-LUX sample representativeness, the sample distributions were compared to the source population distributions according to the stratification criteria (sex, age and district of residence) by classical chi-squared tests. To test whether demographics characteristics and health status affected the participation rate, participants and non-participants were compared by univariate logistic regression analysis. The dependent variable was the participation to the ORISCAV-LUX study while the independent variables were, respectively, prescribed medication, hospitalization (yes or no), cardiovascular health-related medical measures (yes or no), nationality and professional status. Associations were expressed as odds ratios (OR) together with their 95% confidence interval (CI). Age-adjusted ORs were also computed by logistic regression analysis to account for the effect of age. All tests were two-sided, with a P-value of 0.05 or less considered statistically significant.

## Results

### Recruitment process

**Figure 1:**
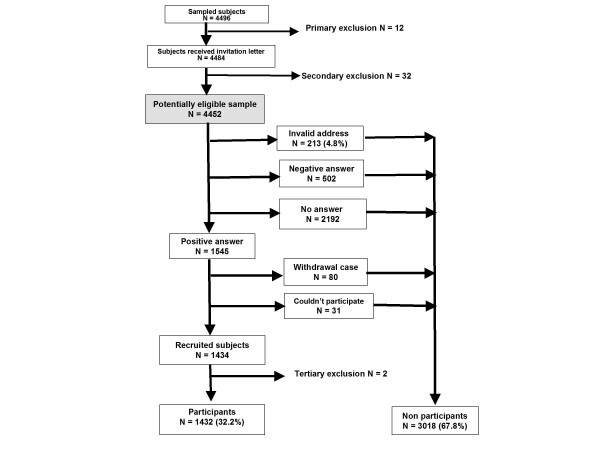
**The ORISCAV-LUX subject's recruitment flowchart**. Primary exclusion before invitation includes 12 addresses of institutionalized subjects. Secondary exclusion after sending the invitation letter includes 32 subjects (pregnant women, mentally ill and deceased cases). Tertiary exclusion from the database after recruitment (> 69 years old).

The ORISCAV-LUX subjects' recruitment process is depicted in Figure 1. Among the 4496 subjects constituting the random stratified sample drawn from the Luxembourg population, 12 were institutionalised and therefore excluded (primary exclusion before sending the invitation letter). After invitation, other 32 cases, including pregnant women (N = 21), severe mentally ill individuals (N = 5), one prisoner and 5 deceased subjects were also excluded before recruitment (secondary exclusion). Thus, a total of 4452 subjects were potentially eligible to take part in the study.

Nearly 5% (213 subjects) were no longer residing at their home address, identified by the post office as "return to sender", were classified as invalid addresses. Further, 502 subjects (11.3%) absolutely refused to participate and were labeled as negative answers; 1545 (34.7%) subjects replied positively and were classified as positive answers; the 2192 (49.2%) individuals who never returned the response-coupon despite 3 invitation letters were considered as "non-respondents". Among initially positive answers, 80 persons refused to continue, were listed as "withdrawal cases", in addition to 31 subjects couldn't come to repeated appointments because of unavailability. The "invalid addresses", "negative answers", "non-respondents", "withdrawal cases" and those how couldn't participate were grouped in a single category called "non-participation", constituting 67.8% of the eligible sample. Finally, a total of 1434 (32.2%) was successfully recruited, hence achieving the prior calculated sample size and the expected response rate. Two participants reached 70 years of age at the time of recruitment were excluded later from the analysis (tertiary exclusion).

Table 1 shows the impact of response-enhancement measures on participation rate. The two postal reminders and the repeated phone contacts enabled further recruitment of 323 (7.3%) and 37 subjects (0.8%), respectively. Thus the participation rate was increased from 24% (1072/4452), then to 31.3% (1395/4452) and finally up to 32.2 (1432/4452) until the end of the study.

**Table 1:** The impact of response-enhancement measures on participation rate (Eligible participants = 4452 subjects)

Response-enhancement measures	Number of positive answers	Participation rate
First invitation	1072	24.1%
Two reminders	323	7.3%
Repeated phone calls	37	0.8%
Total participants	1432	32.2%

A majority of subjects who refused to participate ("negative answers", n = 392; 78.1%) did not indicate the reason. For those who did, the reasons were: lack of time to participate (33 cases), prolonged absence in particular for university students abroad (44 cases), permanent medical control (23 cases), personal reasons (5 cases) and absence of interest in the topic (3 cases).

### Sample representativeness

To assess the representativeness, the recruited sample (1432 participants) was compared to the source population (298,521 individuals) according to the stratification criteria: gender, age category and district of residence. As seen in Table 2, the ORISCAV-LUX sample was representative of the population for gender and district of residence, but not for age categories. This age difference was significant for both men (*P *= 0.0004) and women (*P *= 0.0003). Compared to the source population, the younger age group of 18-29 years was under-represented, whereas adults and elderly were over-represented in the sample.

**Table 2:** Comparison of ORISCAV-LUX participants to the source population by gender, age category and district of residence

Stratification criteria		Source population (N = 298521) % (n)	Participants (n = 1432) % (n)	*P*-value
**Gender**				0.19
	Women	49.64 (148,087)	51.33 (735)	
	Men	50.39 (150,434)	48.67 (697)	
**Age category (years)**				
**Women**				< 0.0001
	18-29	22.38% (33141)	15.6% (115)	
	30-39	25.57%(37865)	24.5% (180)	
	40-49	21.91% (32451)	24.9% (183)	
	50-59	16.41% (24309)	20.3% (149)	
	60-69	13.72% (20321)	14.7% (108)	
**Men**				< 0.0001
	18-29	22.34% (33619)	15.2% (106)	
	30-39	25.72% (38699)	24.5% (171)	
	40-49	22.47% (33811)	27.1% (189)	
	50-59	17.08% (25700)	19.4% (135)	
	60-69	12.37% (18605)	13.8% (96)	
**District of residence**				0.82
	Luxembourg	73.74 (220,116)	72.97 (1045)	
	Diekirch	14.76 (43,956)	15.08 (216)	
	Grevenmacher	11.53 (34,449)	11.94 (171)	

### Comparison of participants and non-participants

The purpose of the non-response analysis was to compare the known demographic and cardiovascular health-related characteristics of participants and non-participants, to reveal potential outcome differences that might indicate a non-response bias. The distribution between participants and non-participants was comparable in terms of the cardiovascular morbidity indicators, including prescribed medications, hospital admission and medical measures (Table 3). Globally, the clinical profile was not dissimilar to that of participants. Concerning prescribed medications, the OR was 1.4 (95%CI: 1.13 - 1.73) for serum lipid reducing agents medication, showing a significant difference between participants and non-participants (10.7% versus 7.89%, *P *= 0.002), but this association disappeared after age adjustment (*P *= 0.12). All prescribed medications in participants were generally similar to those in non participants after age adjustment, except for calcium channel blockers (*P *= 0.04) and for anti-diabetic medications (*P *= 0.049). The odds ratios for hospital admission (OR = 0.54, 95%CI: 0.26 - 1.13) and for cardiovascular health-related medical measures (OR = 1.16, 95%CI: 0.69 - 1.93) did not reveal any significant association with participation, even after age-adjustment, although it was borderline for hospital admission (*P *= 0.05).

**Table 3:** Characteristics of participants and non-participants to the ORISCAV-LUX survey

Characteristics	Participants (N = 1432) % (n)	Non-Participants (N = 3018) % (n)	OR	95% CI	*P*- value	Age adjusted OR	95% CI	*P*-value
Prescribed medication								
Anti-diabetics	2.79 (40)	3.38 (102)	0.82	0.57 - 1.19	0.30	0.68*	0.47 - 1.0	0.049
Anti-thrombotics	7.82 (112)	6.89 (208)	1.15	0.90 - 1.46	0.26	0.99	0.78 - 1.28	0.98
Cardiac therapy	3.56 (51)	3.71 (112)	0.96	0.68 - 1.34	0.81	0.89	0.63 - 1.25	0.48
Antihypertensive drugs	0.56 (8)	0.56 (17)	0.99	0.43 - 2.30	0.98	0.86	0.37 - 2.02	0.73
Diuretics	3.91 (56)	3.64 (110)	1.08	0.78 - 1.50	0.66	0.92	0.66 - 1.29	0.62
Vasoprotective agents	3.14 (45)	2.62 (79)	1.21	0.83 - 1.75	0.32	1.12	0.77 - 1.63	0.55
Beta blocking Agents	6.49 (93)	6.36 (192)	1.02	0.79 - 1.32	0.87	0.85	0.66- 1.12	0.25
Calcium channel blockers	2.37 (34)	3.02 (91)	0.78	0.53 - 1.17	0.23	0.65*	0.43 - 0.98	0.041
Agents acting on the renin-angiotensin system	8.94 (128)	8.68 (262)	1.03	0.83 - 1.29	0.78	0.83	0.65 - 1.05	0.13
Serum lipid reducing agents	10.7 (153)	7.89 (238)	1.40*	1.13 - 1.73	0.0021	1.20	0.95 - 1.52	0.12
Hospitalization	0.63 (9)	1.16 (35)	0.54	0.26 - 1.13	0.10	0.48*	0.23 - 1.0	0.050
Cardiovascular health-related medical measures	1.61 (23)	1.39 (42)	1.16	0.69 - 1.93	0.58	1.06	0.63 - 1.78	0.83
Nationality					< 0.0001			< 0.0001
Luxembourgish	62.08 (889)	54.37 (1641)		Reference			Reference	
Portuguese	13.62 (195)	20.71 (625)	0.58*	0.48 - 0.69	< 0.0001	0.59*	0.49 - 0.71	< 0.0001
French	5.38 (77)	5.77 (174)	0.82	0.62 - 1.08	0.16	0.81	0.61 - 1.07	0.15
Italian	3.56 (51)	4.01 (121)	0.78	0.56 - 1.09	0.14	0.74	0.53 - 1.04	0.084
Belgian	3.77 (54)	3.08 (93)	1.07	0.76 - 1.51	0.69	1.05	0.74 - 1.49	0.78
Other	11.59 (166)	12.06 (364)	0.84	0.69 - 1.03	0.093	0.84	0.68 - 1.03	0.085
Professional status					< 0.0001			< 0.0001
Farmer	1.12 (16)	0.70 (21)	2.15*	1.11 - 4.17	0.024	1.95	1.0 - 3.79	0.050
Employee	31.56 (452)	26.24 (792)	1.61*	1.35 - 1.92	< 0.0001	1.60*	1.34 - 1.91	< 0.0001
State employee	10.82 (155)	7.12 (215)	2.03*	1.59 - 2.6	< 0.0001	1.94*	1.52 - 2.48	< 0.0001
Independent	2.44 (35)	2.49 (75)	1.32	0.86 - 2.01	0.20	1.17	0.77 - 1.79	0.46
Working-class	21.51 (308)	28.79 (869)		Reference			Reference	
Independent intellectual worker	2.37 (34)	1.76 (53)	1.81*	1.15 - 2.84	0.0097	1.66*	1.06 - 2.61	0.030
Other	30.17 (432)	32.90 (993)	1.28*	1.03 - 1.46	0.019	1.22*	1.02 - 1.46	0.030

* Odd ratio is significant when *P*-value < 5% level. The referent category is participation in the ORISCAV-LUX study for dependent variable. The referent category is prescribed medication, hospitalization and medical measures for the independent variables.

The recruited sample was representative of the source population with respect to nationality (*P *= 0.40) (data not shown). However, as seen in Table 3, the participation was significantly associated with the nationality (*P *< 0.0001). The participation rate was lower in Portuguese residents as compared to Luxembourgish (OR = 0.58, 95% CI, 0.48-0.69).

Similarly, the participation was significantly associated with the professional status (*P *< 0.0001). There were 1.12% farmers, 31.56% employees, 10.82% state employees, 2.37% independent intellectual workers, 21.51% from working class among participants against 0.7%, 26.24%, 7.12%, 1.76% and 28.79%, respectively, among non-participants. All ORs were statistically significant, except for the "independent intellectual" category. After age-adjustment, the OR for "farmer" category was no longer significant. Overall, only subjects from the "working class" category were less receptive to the study as compared to the other professional categories.

## Discussion

As non-participation can affect the validity of epidemiological studies, assessment of non-respondents characteristics is important in the critical appraisal of health research. For the same reason, the application of effective strategies to increase response could improve the quality of health surveys and may reduce selection bias. The key objectives of this paper were to analyze the sample representativeness, and to test the null hypothesis that response rate is not influenced by known demographic or health-related characteristics. In addition, it purposed to assess the impact of pro-active measures to increase participation to the national ORISCAV-LUX survey.

In spite of all the forethought and persistence efforts, there are almost always some uncooperative individuals who fail to respond[5]. The two postal reminders enabled further recruitment of 323 subjects, leading to 7.3% increase in response rate. Further, 37 subjects accepted to participate following repeated phone contacts, hence raising the participation rate from 24% up to 32.2% at the end of the study. Whatever the cause, non-respondents may not be a random subgroup and the respondents may not be representative of the parent population. A recent systematic review[28] has examined ways to enhance the response rate, including personalized letters of invitation originating from universities or public authorities, careful layout and user-friendly questionnaires, stamped return envelopes, follow up contacts. Indeed, in the ORISCAV-LUX survey, almost all these measures were applied. Likewise, from our experience, we could suggest that multilingual personnel and documents translations, particularly the questionnaire, to another non-official language(s), augmented considerably the participation of those who prefer answering in their mother language. Moreover, sending by posted mail the cardiologist's feedback regarding clinical and biological results proved to be attractive to the participants. Interestingly, asking the sampled subjects to mention otherwise the reason of refusal was helpful as a prior exploration of non-participation. Though the majority of those who refused (negative answers) did not indicate the cause, several reasons were nonetheless expressed by the subjects, such as: lack of time to participate, prolonged absence of young 18-29 years adults doing their university studies abroad, which was confirmed by the under-participation level in this age group. The recent research developments in the field of population surveys in such small country made the population more solicited and hence keen to participate only to interesting topics. Individuals under permanent medical control seemed to be less disposed and less convinced by the necessity to participate to such surveys. Other personal reasons and absence of interest to the topic were also pointed out.

The main goal of obtaining higher response rates in epidemiological studies is to increase the likelihood of sample representativeness and to decrease "selection bias" [26,29], also denoted "non-response bias" in the text. The sample representativeness analysis have shown that there was no significant difference of the recruited ORISCAV-LUX sample by gender and district of residence as compared with the source population, but the study sample contained a slightly higher proportion of older population and less young adults. This finding was expected as most of the young adults were not available due to their prolonged absence for studying abroad. In addition, the cardiovascular focus of the ORISCAV-LUX study might not be a priority for young age groups who usually enjoy a good health. Conversely, the over-representativeness of the older people might be reasonably due to their perception for the need to clinical check-up, especially when counseling by a cardiologist was provided freely. Therefore, in the ORISCAV-LUX survey, the potential consequence of age-related non-response bias was handled by adjusting the sample for age, by using the most recent available distribution of the Luxembourg's population (STATEC database). According to the research questions, future analyses will be performed after sample weighting to take into account the complex sampling design.

Empirically, a low response rate does not necessarily result in a biased sample or, conversely, a high response rate may yield a biased sample[6,30]. In practice, an "acceptable" or "tolerable" level of non-participation cannot be generalized, because it depends on the study nature, population characteristics and the event under investigation[5]. Although in certain studies a small amount of bias may distort the results, the extent of bias should not necessarily be proportional to the non-response rate[5,6]. In order to address this issue properly, it is necessary to obtain information about the characteristics of non-participants[31].

The literature provides insights regarding the characteristics associated with lower participation, including younger[32] or older age[33,34], female sex[33,34], lower socio-economic status[4,35], lower occupational status[36], non-Western origin[37], comorbidity[38,39], and less favorable lifestyle[40,41]. These findings vary markedly according to the setting, study topic, method and number of contacts, characteristics of the target population and the available data for participants-non participants profile comparison.

It was reported that those who participate in clinical trials and respond to public health surveys are generally healthier and at less risk than those who refuse participation, also called the "healthy participant effect"[1,8,38,42-44], though an example of opposite findings was noticed[45]. To this end, it was important to investigate the morbidity profile of non-participants in the ORISCAV-LUX population. Globally, the morbidity profile-related difference between the participants and non-participants was not significant. Though the participants under lipid-reducing agents were proportionally more numerous than the non-participants, this divergence (dissimilarities of the fractions of the exposed and non-exposed)[35] disappeared after age adjustment. Our results suggest that non-participation in the ORISCAV-LUX survey was not related to the morbidity profile.

Compared to non-participants, participants tended to be older people of Luxembourgish nationality, working as employees or state employees. Across the literature, age seems to have a varying effect[46]; participation either increased with age[37,47,48] or, in the contrary, was higher among the young groups[19,49]. The age difference was probably related to different awareness of the cardiovascular disease problems between young and old people. Such aspects appear to be less attractive to younger age group. In addition, Luxembourg is a small country with limited local higher education possibilities; most young adults of 18-29 years were not available during the study period. Our intensive efforts to fix appointments during the holiday periods increased moderately their participation, but could not surmount their under-representativeness in the sample.

Job grade showed that the probability of participation among state employees was 2.03 times higher than that of those coming from working-classes and 1.94 times after age-adjustment. This finding is close to that observed in most other similar studies in France[46], Great Britain[5,50,51] and United States[52]. The difficulty to get temporary free leave from work may hence limit the attendance of working-class group.

### Strengths and limitations

The present comparative study has major strong points. First, we analyzed non-participation not only according to traditional demographic background but also according to cardiovascular health characteristics. Second, the comparison study was based on reliable data obtained by means of a request to official authorities, without the need of prior research authorization or subject's consent. Third, the national health insurance authorities keep a reasonably complete "medical administrative database" of the general resident population of the country. For health care reimbursement purposes, this database is subject to regular updates, which was confirmed by the relatively low number of invalid addresses. This enabled us to select a national representative sample and to obtain further information on the main cardiovascular health-related conditions. In view of high cost and low or even possibly decreasing participation rates in population surveys, data collected from ongoing routine databanks may represent a good alternative option for health monitoring in the future.

In ORISCAV-LUX, it was not possible to apply a short version of the original self-reported health behaviors questionnaire as an alternative approach to obtain information about the salient outcome indicators (smoking, alcohol consumption, dietary habits and physical activity). For the reason that, after all enhanced efforts to contact and recruit the non-respondents and reluctant cases, it was irrational to re-contact them for an otherwise shorter questionnaire. According to the best of our knowledge, no previous cardiovascular population-based study has reported a similar methodology, based on survey data and national "medical administrative database".

The IGSS data could not supply information about some relevant socioeconomic features, such as the education level, income and marital status, which can be seen as a limitation. Such information would allow investigating the impact of socio-economic differences and its potential bias on the non-participation rate[35]. Therefore, in future analyses, this issue should be interpreted prudently.

## Conclusion

In conclusion, the present article provides an insight regarding the strategies applied to increase response rate, sample representativeness and the characteristics of non participants. The 32.2% participation rate obtained represents the realistic achievable for this type of survey, corresponding to the expected rate, upon which the sample size was calculated. The potential non-response bias of the ORISCAV-LUX data was determined by the degree of similarities between the characteristics of participants and non-participants. Given the absence of divergent cardiovascular health-related profiles, we concluded that the response rate did not invalidate our data and allows generalizing the results for the population. Collectively, these results suggest that pro-active strategies to increase response rate in health surveys may yield unbiased estimates of entire population characteristics.

## Competing interests

The authors declare that they have no competing interests.

## Authors' contributions

AA^(1) ^coordinated the ORISCAV-LUX survey, involved in the study design, collaborated to get the health insurance data, contributed to statistical analysis and results interpretation and drafted the manuscript. NS performed the statistical analysis and contributed to results interpretation. SC gave methodological advice on the present study design and statistical analysis. M-LL participated in the present study design, coordinated between the authorities to get the health insurance data, supervised all the study process and discussed the statistical results. AA^(2) ^contributed to the critical revision of the manuscript and intellectual content. MG provided expertise and oversight throughout the process. All authors read and approved the final version.

## Pre-publication history

The pre-publication history for this paper can be accessed here:

http://www.biomedcentral.com/1471-2288/10/80/prepub
